# Diesel production from lignocellulosic feed: the bioCRACK process

**DOI:** 10.1098/rsos.171122

**Published:** 2017-11-15

**Authors:** K. Treusch, J. Ritzberger, N. Schwaiger, P. Pucher, M. Siebenhofer

**Affiliations:** 1 BDI – BioEnergy International AG, Parkring 18, 8074 Raaba-Grambach, Austria; 2 Institute of Chemical Engineering and Environmental Technology, Graz University of Technology, Inffeldgasse 25/C, 8010 Graz, Austria

**Keywords:** bioCRACK, biofuel, liquid phase pyrolysis, refinery integrated

## Abstract

The bioCRACK process is a promising technology for the production of second generation biofuels. During this process, biomass is pyrolized in vacuum gas oil and converted into gaseous, liquid and solid products. In cooperation with the Graz University of Technology, the liquid phase pyrolysis process was investigated by BDI – BioEnergy International AG at an industrial pilot plant, fully integrated in the OMV refinery in Vienna/Schwechat. The influence of various biogenous feedstocks and the influence of the temperature on the product distribution in the temperature range of 350°C to 390°C was studied. It was shown that the temperature has a major impact on the product formation. With rising temperature, the fraction of liquid products, namely liquid CHO-products, reaction water and hydrocarbons, increases and the fraction of biochar decreases. At 390°C, 39.8 wt% of biogenous carbon was transferred into a crude hydrocarbon fractions. The type of lignocellulosic feedstock has a minor impact on the process. The biomass liquefaction concept of the bioCRACK process was in pilot scale compatible with oil refinery processes.

## 1. Introduction

According to the adoption of the Paris Agreement in 2015 the global average temperature increase is to be kept significantly below 2°C above the preindustrial level. This target has to be achieved by reducing greenhouse gas emissions without threatening food production [1]. Second generation biofuels based on lignocellulose will play a sustainable key role for future fuel production from renewable feedstock. Main lignocellulose-conversion technologies are: indirect liquefaction via gasification and methanol synthesis or Fischer–Tropsch synthesis; pyrolysis-based direct liquefaction with subsequent hydrodeoxygenation; upgrading of residues from solvent-based pulping processes and biotechnological treatment of biomass [2]. Gasification methods such as the Choren technology and the successor Linde Carbo-V® technology [3] require extensive gas treatment in order to remove impurities from crude gas for subsequent processes like Fischer–Tropsch synthesis [4]. Particles have to be removed and sulfur content reduced by gas cleaning concepts, e.g. the low-temperature Rectisol process using methanol as scrubbing agent [5]. Bioethanol from fermentation of cellulose can be admixed to gasoline. However, bioethanol-based biofuels are faced with several unfavourable properties like different density, corrosiveness, low calorific value, low boiling point and miscibility with water, to mention just a few [6]. Pyrolysis of biomass yields in the production of pyrolysis oil, biochar and pyrolysis gas, which can be further upgraded to biofuels [7]. Crude products from dry pyrolysis of lignocellulose need extensive pretreatment for further processing due to coal particle load and the high viscosity of pyrolysis oil. The solids content varies between 0.3% and 3% [8].

Liquid phase pyrolysis (LPP) [9,10], as applied in the bioCRACK process [11], is a promising alternative technology for fuel production from lignocellulosic biomass. The acronym ‘bioCRACK’ was generated from the words ‘biomass’ and ‘cracking’, as the biomass is cracking the carrier oil. BioCRACK is a registered word and design mark. In liquid phase pyrolysis, biomass is thermally treated in a heat carrier oil, e.g. vacuum gas oil (VGO), a side product of crude oil refining. The process is operated in the temperature range between 350°C and 390°C. Due to elevated operation temperature, VGO is partially cracked during pyrolysis, an advantageous side effect of biomass pyrolysis. Biomass is converted into pyrolysis gas, biochar and liquid products. The homolytic degradation process of biomass during pyrolysis is slightly modified by the influence of VGO. Repolymerization reactions are reduced and particles are held back in the heat carrier. The liquid products partially dissolve in vacuum gas oil and may be processed in the refinery without further treatment, achieving a direct transfer of biogenous carbon into VGO. The bioCRACK process was operated in pilot scale (100 kg h^−1^ biomass) at OMV refinery in Vienna/Schwechat [12]. Vacuum gas oil limits the pyrolysis temperature to about 400°C because of its boiling range between 365°C and 530°C [13]. This allows process operation at ambient pressure which simplifies construction and significantly decreases investment costs. Kumar *et al*. [14] have described a related process with a pressure of at least 50 bar and a reaction time of at least 15 min. Nevertheless, the bioCRACK process is the first direct biomass liquefaction process that has already been operated in pilot scale in a petrol refinery. Compared to other biomass liquefaction technologies, the concept is very simple [3–5,7].

### 1.1. BiomassPyrolysisRefinery

For an economic operation of the bioCRACK pyrolysis process, a high recovery of all product streams is obligatory. The upgrading of the two main side streams biochar and pyrolysis oil is part of the BiomassPyrolysisRefinery concept [15,16,17]. Feiner [18] investigated the liquefaction of biochar, Pucher [19] the hydrodeoxygenation of LPP oil.

Experiments for the liquefaction of biochar [20] were performed in a stirred 450 ml batch reactor between 370°C and 450°C at 180 bar. In order to avoid polymerization reactions [21], hydrogen was provided with tetralin as a hydrogen donor. Biochar conversion of 84 wt% and an oil yield of 72 wt% could be achieved. Hydrodeoxygenation of LPP oil [22] was investigated at 250°C and 100 bar and at 300°C and 150 bar in a batch reactor. Pyrolysis oil from the bioCRACK process has a higher water content than pyrolysis oil from flash pyrolysis [23]. This results in a lower viscosity. In LPP oil, no particles are present, as they are held back together with the biochar by the VGO. This facilitates the handling of pyrolysis oil. An oil yield of up to 56 wt% was observed.

## 2. Material and methods

Analytical methods, feedstock and process parameters will be discussed in this section.

### 2.1. Feedstock material

Lignocellulosic feedstock was spruce wood, beech wood, miscanthus and wheat straw. All lignocelluloses were provided in pelletized form. The pellets were milled on-site with a mechanical mill. For temperature study, spruce wood was used as lignocellulosic feed. Spruce wood was provided in ENplus A1 and DINplus accredited pellets from RZ Pellets GmbH in Bad St Leonhard, Austria. Beech wood and wheat straw were provided by FAIR Holz in Leopoldshöhe, Germany. Miscanthus was provided by TD Zorn GmbH in Heidenrod–Zorn, Germany. An elemental analysis of the feedstock is shown in table 1. Lignocellulosic biomass contains up to 50% oxygen [24]. This oxygen has to be removed in order to produce hydrocarbons with fuel quality. The carbon content of the wood samples varies between 43.3 and 50.1%.

**Table 1.** Elemental analysis of feedstock material.

	carbon [wt%]	hydrogen [wt%]	nitrogen [wt%]	balance [wt%]
spruce wood	50.1	6.3	0.04	43.5
beech wood	45.3	6.3	0.09	48.3
miscanthus	43.3	6.4	0.14	50.2
wheat straw	45.1	6.0	0.58	48.4
VGO	86.31	12.26	0.55	0.88

Liquid phase pyrolysis product formation is not significantly dependent on the particle size of the biogenous feedstock in the range between 1 mm and 1 cm [9]. The elemental composition of VGO is shown in table 1. Vacuum gas oil has a boiling range between 365°C and 530°C [13]. The VGO used for the bioCRACK had a boiling range between 300°C and 530°C.

### 2.2. Analytical methods

The elemental analyses of all streams were characterized by a vario MACRO CHN-analyzer from ‘Elementar Analysensysteme GmbH’. The water content of pyrolysis oil was determined by a gas chromatograph, type Agilent 7890A, with a TCD detector and a HP-INNOWAX column, 30 m×0.53 mm×1 µm. The water content of the oil fraction was determined by Karl-Fischer-titration with a Schott Titro Line KF-Titrator and a Hydranal titration reagent. The boiling range of the oil fractions was determined by a gaschromatograph, type Agilent 7890A, with an FID-detector and the Restek-column MXT-2887, 10 m×0.53 mm×2.65 µm. Density and viscosity were measured with a digital viscometer, SVM 3000, of Anton Paar GmbH. The content of biogenous carbon (^14^C) was determined by the external laboratory Beta Analytic Limited, SO/IEC 17025:2005 accredited, in Miami, FL, via acceleration mass spectrometry.

### 2.3. The bioCRACK pilot plant

**Figure 1.**
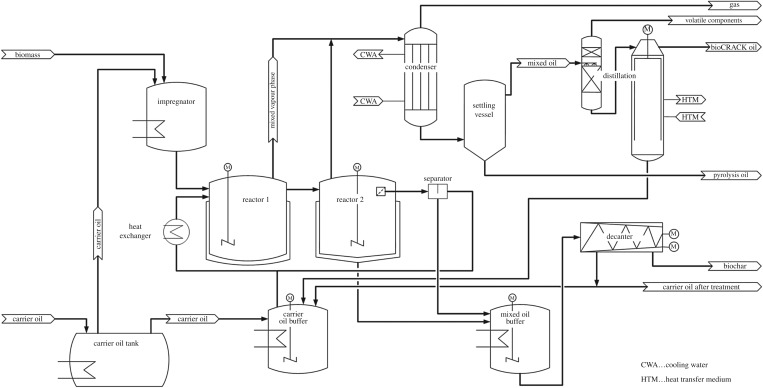
Integration of the bioCRACK process in a refinery [11].

**Figure 2.**
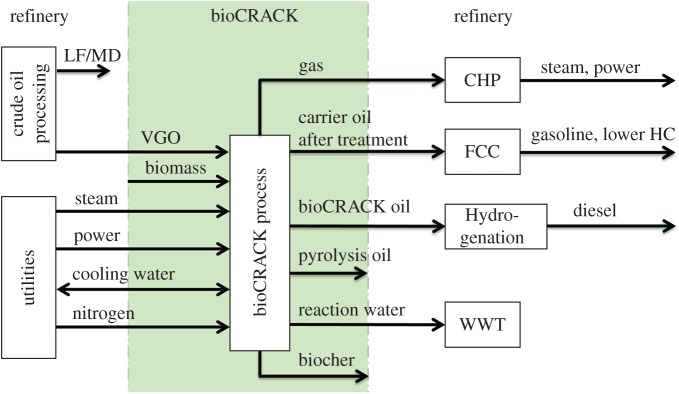
Scheme of the bioCRACK process [16].

The bioCRACK pilot plant with a nominal biomass capacity of 100 kg h^−1^ and 600 kg h^−1^ VGO was in continuous operation for two years. The mass ratio of VGO to biomass was 6. A scheme of the bioCRACK process is shown in figure 1. Biomass is impregnated with VGO in a first vessel and then fed in reactors 1 and 2 together with additional recycle-VGO. The reaction takes place at 350–400°C and ambient pressure. Biogenous and fossil vapours are condensed and separated in two steps, obtaining the aqueous pyrolysis oil fraction, the non-polar bioCRACK oil fraction and high boiling heat carrier residues. Biochar and heat carrier are separated in a decanter. Figure 2 shows the integration concept of the bioCRACK process in an existing refinery [11], as practised during operation of the pilot plant. Utilities, such as steam, power, cooling water and nitrogen, can be used from the refinery. Gaseous products generate electricity and/or steam. The reaction products can be upgraded in existing refinery facilities.

## 3. Results and discussion

**Figure 3.**
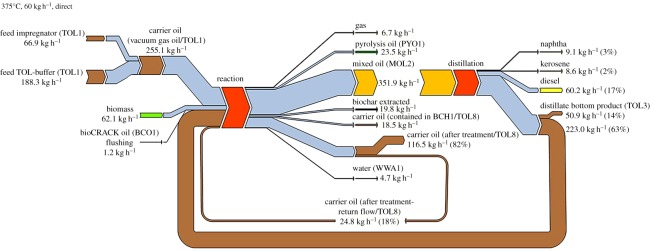
Mass balance of the bioCRACK process (375°C, spruce wood).

The mass balance of the liquid phase pyrolysis process at 375°C with the feedstock spruce wood is shown in figure 3. Process operation is 5 days, balance period is about 36 h. The carrier oil feed is composed of the oil from the impregnator and the carrier oil buffer. To minimize the consumption of VGO, the bottom product of the distillation as well as the spent VGO are recycled. The reaction products consist of non-condensible gases (at ambient temperature and pressure), pyrolysis oil, mixed oil, pyrolysis char, spent VGO and the water-content of the biomass. The water formed during LPP and the feed moisture are discharged with the pyrolysis oil. The amount of dry biochar is determined analytically through extraction of VGO residues with hexane in laboratory scale. The composition of these product streams is shown in table 2. The mixed oil is distilled to obtain bioCRACK oil (naphtha, kerosene, diesel) and a bottom product. The MIX-buffer contains spent VGO and biochar and is fed to the decanter. The bioCRACK oil feed is used for washing purposes between experiments.

**Table 2.** Product characterization in dependence of the pyrolysis temperature.

[wt%]	biochar			pyrolysis oil			mixed oil			carrier oil after treatment			gas		
reaction temperature [°C]	350	375	390	350	375	390	350	375	390	350	375	390	350	375	390
C [wt%]	77.5	80.9	80.9	25.8	25.6	26.6	85.3	84.9	84.3	86.2	86.6	86.3	—	—	—
H [wt%]	5.3	5.4	5.4	9.2	9.4	9.2	12.3	12.3	12.1	12.2	12.1	11.8	—	—	—
N [wt%]	0.2	0.3	0.3	0.3	0.4	0.4	0.4	0.4	0.5	0.5	0.5	0.6	—	—	—
residue [wt%]	16.9	13.5	13.4	64.7	64.6	63.8	2.0	2.4	3.2	1.2	0.9	1.3	—	—	—
water [wt%]	—	—	—	50.2	51.9	50.2	—	—	—	—	—	—	—	—	—
^14^C [wt%]	88.9	84.1	79.4	100	100	100	4.9	6.7	3.4	1.7	2.1	1.7	—	—	—
CO [wt%]	—	—	—	—	—	—	—	—	—	—	—	—	33.9	34.4	33.4
CO_2_ [wt%]	—	—	—	—	—	—	—	—	—	—	—	—	61.5	55.0	53.4
CH_4_ [wt%]	—	—	—	—	—	—	—	—	—	—	—	—	4.6	10.6	13.2

### 3.1. Influence of the reaction temperature

**Figure 4.**
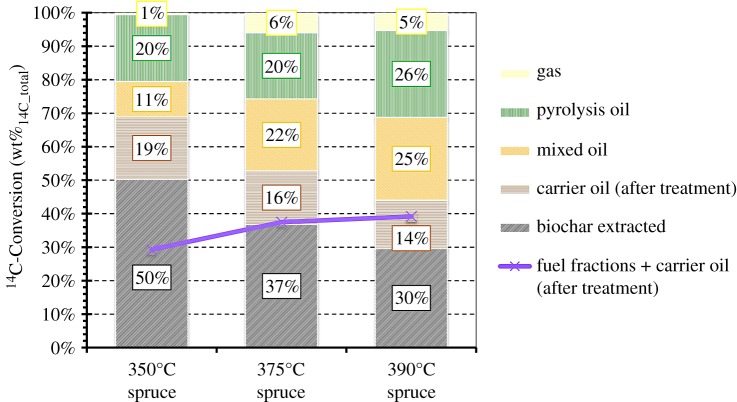
Bio-carbon transfer as a function of the pyrolysis temperature.

The reaction temperature has a major impact on the product distribution of pyrolysis processes. The higher the temperature, the more gas and the less biochar is produced, whereas liquid products show a maximum at 500°C (fast pyrolysis processes) [25]. As shown in figure 4, lignocellulose is transferred into biochar, hydrocarbons, pyrolysis oil and gaseous products during liquid phase pyrolysis. Elevated temperature leads to a decreasing amount of biochar and rising liquefaction. At temperatures below 385°C, biochar is the main reaction product, and liquid CHO products above 385°C.

The balance of biogenous carbon is in accordance with the mass balance. With increasing temperature, the biochar yield decreases and more liquid products are formed. The transfer of biogenous carbon into the fossil oil fraction rises with increasing temperature. The transfer of biogenous carbon into hydrocarbon fraction increases from 29 wt% at 350°C to about 40 wt% at 390°C. The difference in ^14^C balance compared to the mass balance is caused by the different carbon content of the individual products. Whereas biochar has a carbon content of 81% and hydrocarbons about 85%, the carbon content of pyrolysis oil amounts to 26%, as shown in the elemental analysis in table 2.

**Figure 5.**
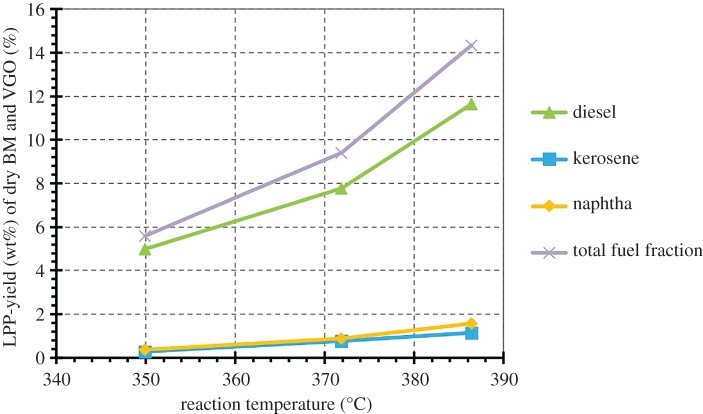
Fuel yield as a function of the pyrolysis temperature.

Through simulated distillation, the bioCRACK oil can be analytically separated in diesel, kerosene and naphtha. With increasing temperature, more fuel gets produced due to VGO cracking. The total amount of fuel fraction based on biomass (BM) and VGO as a function of reaction temperature is shown in figure 5. Although the ^14^C amount of all fractions rises with temperature, the impact on the diesel fraction deviates strongly. The total amount of fuel is about 5–6% at 350°C. It rises up to 14–15% at 386°C.

### 3.2. Influence of the biomass feedstock

The bioCRACK process is not very sensitive to different lignocellulosic feedstock. The transfer of biogenous carbon into the fuel fraction of beech wood and wheat straw is similar to the data for spruce wood. The transfer of biogenous carbon into the non-condensable gaseous products is higher for the feedstock wheat straw. This results in a lower CHO-content of the pyrolysis oil (about one third compared to 50% for spruce or beech wood). The CHO-content of pyrolysis oil from miscanthus amounts to about 40%. Caused by the higher ash content of miscanthus and wheat straw, more biochar, but with a lower heating value, is formed. There is no significant difference concerning the power consumption.

### 3.3. Product characterization

**Figure 6.**
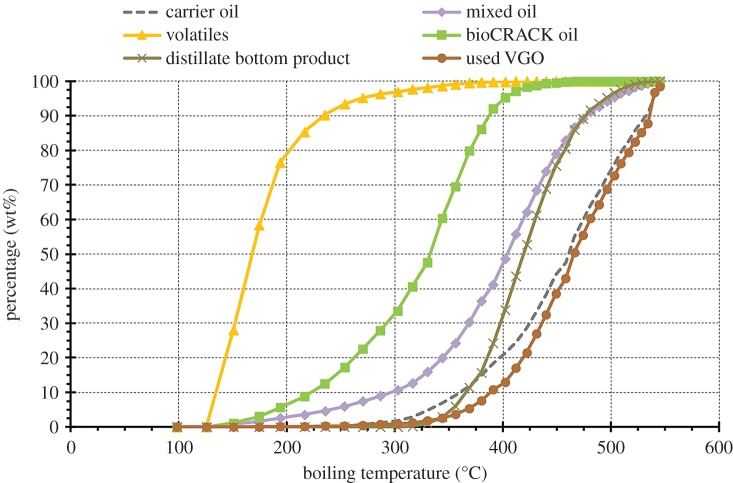
Boiling range of the liquid feed and products (375°C, spruce wood).

The boiling range of the carrier oil and products is shown in figure 6. The boiling range of spent VGO does not differ from fresh VGO fed to the reactor. The amount of biogenous carbon in the spent VGO is 2–3%. Caused by the small difference in the boiling range, the spent VGO can be fed directly into the refinery FCC. The mixed oil is distilled to yield volatiles, bioCRACK oil and a bottom product. The boiling curve of the bioCRACK oil lies between the bottom product and the volatiles.

The main component of pyrolysis oil is water. It mainly consists of acids, ketones, aldehydes, sugars, phenols and an organic matrix from sugar and lignin derivatives and other substances [22]. A specific extraction is very complex due to the high number and low content of individual components [23]. The composition and characterization of pyrolysis oil is shown in table 3. Caused by the high oxygen content, a direct admixture to fuels is not possible. Pyrolysis oil is very corrosive and therefore needs to undergo further upgrading.

**Table 3.** Characterization of liquid phase pyrolysis oil.

water content	[wt%]	49.6
heating value	[MJ kg^−1^]	9.2
density	[kg m^−3^]	1090
kinetic viscosity	[mm^2^ s^−1^]	3.9
biogenous carbon	[wt%]	100
carbon	[wt%]	25.6
hydrogen	[wt%]	9.2
oxygen	[wt%]	64.9
nitrogen	[wt%]	<1

### 3.4. Further product processing

The products can be upgraded with existing facilities of oil refineries, where raw fuel and VGO would be processed anyhow. These upgrading facilities are the fluid catalytic cracker (FCC) and hydrogenation reactors for fuel production with a biogenous content [11].

Before hydrogenating in laboratory scale, bioCRACK oil was treated by distillation to produce the fractions light ends (less than 175°C), kerosene (175–225°C), gas oil (225–350°C) and bottom products (more than 350°C). Afterwards, kerosene and gas oil were hydrogenated in a hydrotreater with a feed rate of 65 ml h^−1^ at 41 bar and 360°C. The required hydrogen flow of 200–220 Nm^3^ m^−3^ for bioCRACK oil hydrogenation is higher than that for crude raw fuel with about 135 Nm^3^ m^−3^. Also the temperature is higher. This is caused by the higher oxygen content of bioCRACK raw fuel. The density of the hydrogenated gas oil is slightly off the standard for diesel. To achieve the standards, the gas oil has to be fractionated between 200°C and 350°C. After that, the gas oil achieves the quality of diesel according to EN590, as shown in table 4.

**Table 4.** Quality of bioCRACK diesel before and after hydrotreatment compared to EN 590.

parameter	untreated raw diesel	after hydrotreatment	EN 590
density (15°C) [kg m^−3^]	868	833	820–845
viscosity (40°C) [mm^2^ s^−1^]	2.53	n.a.	2–4.5
Cetan	44	53	>51
C/H/O [wt%]	85/13/2	86/14/0	n.a.
volatile <350°C [wt%]	83	86	>85% (v/v)
sulfur [mg kg^−1^]	177	3	<10

The heavy oil fraction is cracked in an FCC. The feed is preheated in a tubular furnace close to boiling, which is approximately between 260°C and 320°C. The feed evaporates instantaneously when getting in contact with the catalyst and is cracked [26].

Berchtold *et al*. [26] investigated four different case studies to survey the influence of different VGO pretreatment and biomass feedstock on the treatment in the FCC. The case studies included: spruce wood and VGO without hydrotreatment, spruce wood and VGO hydrotreated after LPP, spruce wood and VGO hydrotreated prior to LPP, and straw and VGO without hydrotreatment. In case studies without hydrotreatment, more coke was formed. Preceding hydrotreatment reduces coking reactions. The biogenous feedstock straw reduces the yield of fuel due to coking reactions and higher residue. However, the outcome of all case studies did not show significant deviations. It was shown that high conversion efficiency for all performed case studies due to the very low oxygen content of the processed heat carrier oil was achieved. The FCC therefore can be used for cracking of spent VGO with biogenous content from the bioCRACK process without any major modifications of the FCC plant design.

## 4. Summary and conclusion

In pilot scale operation, up to 40 wt% of the biogenous carbon yielded in hydrocarbon refinery intermediates and fuel fractions. Up to now, the bioCRACK process is the first technology for direct biomass liquefaction integrated in an oil refinery process. However, this process has so far only been practised in pilot scale, the next step would be a demonstration plant. For an economic operation, the bioCRACK process would have to be investigated in industrial scale. The temperature has a major impact on the composition and distribution of LPP products. Higher reaction temperature leads to higher liquid product yield and lower solids yields. The transfer of biogenous carbon into the fuel fraction rises with temperature. The type of lignocellulose has a minor impact on the process. Compared to spruce wood, the main feedstock, beech wood shows no significant difference, while miscanthus and wheat straw lead to lower CHO yield and a higher biochar production with a lower heating value due to the higher ash content. The hydrocarbons can be upgraded in existing refinery facilities with some adaptions. The upgrading of the main side streams, biochar and pyrolysis oil, has been investigated in laboratory scale with promising results.

## Data accessibility

All the performance data are provided in the main text. There are no additional data associated with this submission.
